# Internet addiction and psychological inflexibility in young adults: a parallel mediation analysis of alexithymia facets within an I-PACE framework

**DOI:** 10.3389/fpsyt.2026.1922964

**Published:** 2026-08-31

**Authors:** Daniele Mollaioli, Clara Lombardo, Barbara Salvo, Maria Imbesi, Luana Sorrenti, Federica Zuccalà, Carmela Mento

**Affiliations:** 1 Department of Clinical and Experimental Medicine, University of Messina, Messina, Italy; 2 Department of “Health Sciences”, University Magna Graecia of Catanzaro, Catanzaro, Italy; 3 Department of Human Pathology, Medical Oncology Unit, Polyclinic Hospital, Messina, Italy; 4 Psychiatric Unit, Polyclinic Hospital, Messina, Italy; 5 Department of Biomedical and Dental Sciences and Morphofunctional Imaging, University of Messina, Messina, Italy

**Keywords:** alexithymia, emotion regulation, internet addiction, psychological inflexibility, young adults

## Abstract

**Introduction:**

Internet Addiction (IA) is a rising issue among young adults and is associated with emotional, relational, and cognitive difficulties. In line with the I-PACE model, this study aimed to explore the mediating role of the three dimensions of alexithymia (Difficulty Identifying Feelings—DIF; Difficulty Describing Feelings—DDF; and Externally-Oriented Thinking—EOT) in the relationship between IA severity and psychological inflexibility.

**Method:**

The study involved 224 young Italian adults (Mage = 23.76, SD = 5.82) with mild and moderate levels of IA, defined by a total score on the Internet Addiction Test (IAT) of ≥ 30. A parallel multiple mediation analysis was conducted using the PROCESS macro for R.

**Results:**

The results showed that the severity of IA was positively associated with all dimensions of alexithymia. The mediation analysis revealed two significant indirect pathways: DIF negatively mediated the relationship between IA and psychological inflexibility, while EOT showed a positive mediation effect. In contrast, DDF did not produce any significant indirect effects.

**Discussion:**

Overall, the results suggest that different dimensions of alexithymia contribute in different ways to the relationship between IA and psychological inflexibility. The observed association may reflect an apparent flexibility rather than actual adaptive capacity, highlighting the need for longitudinal studies to further clarify the underlying mechanisms.

## 1 Introduction

The rapid spread of digital technologies has profoundly changed the way people live, communicate and work (1), but it has also created new challenges. Among them, Problematic Internet Use (PIU) and Internet Addiction (IA) describe difficulties in controlling online activity that are associated with negative consequences in daily life (2–4). Although PIU and IA reflect partially overlapping, they conceptually represent two distinct constructs (5); this distinction is important because generalized Internet addiction is not formally recognized as an independent diagnosis in major classificatory systems, whereas PIU/PUI is increasingly used to denote maladaptive internet-related behaviors without implying a specific disorder (6–8). Nevertheless, we retain the term IA here because it remains the terminology adopted by the Internet Addiction Test (IAT), one of the most widely used instruments in this field (4, 9, 10).

This phenomenon has attracted increasing attention in research on emotional and cognitive difficulties, often associated with compulsive internet use to regulate emotions and mood (11, 12), and with irritability, anger, and distress when access is limited (13, 14).

Over time, these difficulties can undermine social, emotional and occupational functioning. (3, 9, 15, 16). Adolescents and young adults appear particularly vulnerable to the development of IA due to the widespread and portable nature of digital devices (17), with comorbid conditions like depression and ADHD amplifying these adverse effects (18), and the role of emotion regulation strategies, in combination with specific affective temperaments (19).

To explain the mechanisms underlying these associations, the present study draws on the Interaction of Person-Affect-Cognition-Execution (I-PACE) model, a process-based framework that conceptualizes addictive internet-related behaviors as the result of dynamic interactions among person-level vulnerabilities (P), affective-cognitive responses (A-C), and executive-control deficits (E). (20, 21). Within this architecture, predisposing vulnerabilities at the P level actively interact with A-C processes and E-level deficits to maintain compulsive internet use over time (22).

The I-PACE model has been increasingly applied to specific internet-use disorders and other addictive behaviors, supporting its usefulness as a general framework for behavioral addiction research (23–26).

Within the I-PACE framework, predisposing affective vulnerabilities at the Person (P) level play a central role in the development of IA. Among these, alexithymia, characterized by difficulties in recognizing, distinguishing and verbally describing one’s own and others’ emotional states (27), has been extensively linked to IA among both adolescents and university students, where it may operate as an emotional avoidance strategy that exacerbates emotional and social difficulties (28–31). Mapped onto the I-PACE model, this deficit in interoceptive awareness may be associated with a greater reliance on the digital environment as a compensatory coping strategy, wherein Internet use serves as an external, albeit maladaptive, means to modulate affective distress and circumvent offline emotional processing demands (32–34).

At the Execution (E) level of the I-PACE model, psychological inflexibility represents a key cognitive-emotional mechanism sustaining compulsive Internet-related behaviors. Psychological flexibility allows people to cope with stress, maintain balance across important life domains, and remain open and committed to value-congruent behaviors (35, 36). Conversely, psychological inflexibility, characterized by the rigid dominance of psychological reactions over personal values or situational demands in guiding actions (37), has been associated with greater PIU and IA (16, 38) and may reflect a key cognitive–emotional mechanism underlying IA, operating through its association with elevated depression, anxiety, hostility, and interpersonal sensitivity (15).

Individuals with alexithymia generally present lower levels of psychological flexibility (39, 40), suggesting that the P-level and E-level vulnerabilities identified within the I-PACE model are not independent but mutually reinforcing.

Despite growing evidence linking IA to both alexithymic traits and psychological inflexibility, it remains unclear whether the three alexithymia facets operate as distinct parallel mediators in the relationship between IA severity and psychological inflexibility. The present study addresses this gap by testing a parallel multiple mediation model in an Italian adult sample with confirmed IA. This approach is relevant because it clarifies whether different alexithymia facets contribute in distinct ways to the link between IA and psychological inflexibility, thereby extending previous work that treated alexithymia as a unitary construct. Based on theoretical and empirical considerations, three *a priori* hypotheses were formulated:

**(H1)** Difficulty Identifying Feelings (DIF) was expected to exert a significant positive indirect effect on psychological inflexibility, given that the inability to identify one’s emotional states represents a core P-level vulnerability within the I-PACE model that is theoretically linked to reduced introspective capacity and, by extension, to greater experiential avoidance. (31, 41)
**(H2)** Externally-Oriented Thinking (EOT) was expected to exert a significant positive indirect effect on psychological inflexibility, as the externally-oriented cognitive style characteristic of this facet aligns with the A-C-level mechanisms of attentional disengagement from internal states and reduced perspective-taking. (39, 42)
**(H3)** Difficulty Describing Feelings (DDF) was expected to yield a weaker or non-significant indirect effect, given that the interpersonal-communicative dimension of alexithymia is less directly implicated in the intrapersonal regulatory processes captured by the AAQ-II (27, 30).

## 2 Materials and methods

### 2.1 Study design and population

Data were collected between 2024 and 2025 through an online survey sent to institutional or other professional mailing lists, posted on social networks and via web advertising.

Participants received documented details regarding the objectives, methodologies and anticipated advantages of the study; the submission of the survey was considered informed voluntary consent. The study was conducted with subjects complying with the Declaration of Helsinki and was approved by the local ethics committee of Centre for Psychological Research and Intervention (Ce.R.I.P.) for studies involving humans. Inclusion criteria were: (1) age between 18 and 30 years; (2) fulfilling all study questionnaires; (3) presence of Internet Addiction, defined as a total IAT score ≥ 30, in accordance with the scoring guidelines provided by original and Italian validation studies (10, 43). Among 257 participants, 33 were excluded due to not meeting inclusion criteria. A final sample of 224 subjects was considered for statistical analysis.

### 2.2 Instruments

A demographic questionnaire, including age, gender, and school of origin, was used to collect participants’ basic demographic information, while different psychological instruments were administered to assess the variables under study. *Internet Addiction Test* (10), was used to measure Internet addiction. The scale consists of 20 items rated on a 5-point Likert scale (1 = “Never,” 5 = “Always”) and investigates several aspects related to problematic Internet use, including: emotional and cognitive concerns, Internet-related social consequences, loss of control and interference with daily duties. The *Italian version of Internet Addiction Test* (43) identified a two main factor structure: emotional and cognitive concerns and loss of control. The IAT demonstrated excellent internal reliability (Cronbach’s alpha between 0.83 and 0.86) and convergent validity with other scales of problematic Internet use (10, 43).

The degree of psychological flexibility was assessed through the *Acceptance and Action Questionnaire-II* (AAQ-II, 44). The questionnaire consists of 7 items, rated on a 7-point Likert scale (1 = “Never true,” 7 = “Always true”), with higher scores indicating greater psychological inflexibility. The Italian validation confirmed the unidimensional structure and good internal reliability (Cronbach’s alpha = 0.83). The instrument demonstrated convergent validity with measures of anxiety, depression, and psychological well-being and has proven to be an effective tool for investigating the role of psychological flexibility in various clinical and research settings (45).

Finally, *Toronto Alexithymia Scale-20* (TAS-20) was used to evaluate alexithymia (34, 42). The scale consists of 20 items rated on a 5-point Likert scale (1 = “Strongly disagree,” 5 = “Strongly agree”). The scale is structured around three main factors: difficulty in identifying feelings (DIF), difficulty in describing feelings (DDF), outward-oriented thinking style (EOT). The TAS-20 demonstrated high internal reliability, with Cronbach’s alphas above 0.80, and construct validity confirmed in different populations and cultural contexts (34). The *Italian version of the TAS-20* (46)retained the three-dimensional construct and has proven to be an effective tool for assessing alexithymia in both clinical and research settings and in various samples of adults and adolescents (47, 48).

### 2.3 Statistical analysis

All statistical analyses were conducted using the R software (49). Specifically, the PROCESS macro for R Version 5.0 (50) was utilized to investigate the hypothesized parallel mediation. Model 4 was applied to test a multiple parallel mediation model designed to evaluate whether specific facets of alexithymia mediate the relationship between IA and psychological inflexibility.

The total score of the IAT was entered as the independent variable (X), while the total score of the AAQ-II served as the dependent variable (Y). The three subscales of the Toronto Alexithymia Scale—DIF, DDF, and EOT —were simultaneously entered as parallel mediators (M1, M2, M3). To control for potential confounding effects and isolate specific relationships under investigation, several socio-demographic and behavioral variables were included in the model as covariates: gender, education level, time spent on social media, social avoidance, time spent at home, time spent outside, social difficulties, and relationship status.

Furthermore, to ensure the robustness of the findings and account for potential heteroscedasticity in the data, a heteroscedasticity-consistent standard error and covariance matrix estimator (HC4) was employed for all regression models. The significance of the indirect (mediating) effects was evaluated using a bootstrapping procedure with 5,000 resamples to generate 95% percentile confidence intervals (CIs). An indirect effect is considered statistically significant if the corresponding 95% CI does not include zero.

Additionally, a comprehensive set of regression diagnostics was performed to verify model assumptions and assess data quality. Multicollinearity among the predictors was evaluated using Tolerance and the Variance Inflation Factor (VIF). Heteroscedasticity was formally tested using both the standard and robust variants of the Breusch-Pagan test. Residual analysis was conducted to inspect the distribution of errors, evaluating skewness and kurtosis alongside a Bonferroni-corrected significance test for studentized residuals to screen for extreme outliers.

Finally, a *post-hoc* power analysis conducted with the *pwr* package in R (51) indicated that, with N = 224, 12 predictors, α = .05, and power = .80, the minimum detectable effect size was f² = 0.081, falling within the small-to-medium range according to Cohen’s conventions. (52).

## 3 Results

The final sample was composed by 224 participants, with a mean age of 23.76 (SD = 5.82) and constituted by females in majority (75.4%). All demographic characteristics of the study sample were summarized in **Table 1**.

**Table 1:** Demographic and behavioral characteristics of the sample (N = 224).

Variable	Category	N	%
Gender			
	Male	55	24.6
	Female	169	75.4
Age		23.76 ± 5.82	
Relationship status			
	Engaged	120	53.6
	Single	104	46.4
Education level			
	Undergraduated	138	61.6
	Graduated	86	38.4
Social media use			
	Up to 3 hours	89	39.8
	About 4–5 hours	112	50.0
	Always online	23	10.3
Presence of social avoidance			
	Yes	85	37.9
	No	139	62.1

**Table 2** reports the descriptive statistics, distribution indices, and internal consistency coefficients for the study variables. 159 (71.0%) were classified as having mild Internet addiction and 65 (29.0%) as having moderate-to-severe Internet addiction. Regarding alexithymia severity, 138 participants (61.6%) fell in the non-alexithymic range, 48 (21.4%) in the possible alexithymia range, and 38 (17.0%) in the alexithymia-present range.

**Table 2:** Descriptive statistics and reliability indices for the study variables.

Variable	Mean	SD	Kurtosis	Asymmetry	Reliability
Internet Addiction (IAT)	44.875	9.215	-0.292	0.572	0.781
Psychological Inflexibility	31.339	9.100	-0.756	-0.244	0.877
Difficulty Identifying Feelings	17.210	6.766	-0.942	0.355	0.866
Difficulty Describing Feelings	14.263	4.730	-0.612	0.100	0.758
Externally-Oriented Thinking	16.361	4.591	-0.320	0.390	0.709

**Table 3** shows the Pearson correlation matrix among the study variables. Internet addiction was positively associated with Difficulty Identifying Feelings, Difficulty Describing Feelings, and Externally-Oriented Thinking, and negatively associated with psychological inflexibility (all p <.001, except IAT–DDF: p = .0154). Psychological inflexibility was strongly and negatively related to Difficulty Identifying Feelings and Difficulty Describing Feelings (both p <.001), whereas its association with Externally-Oriented Thinking was negligible and non-significant (p = .7355).

**Table 3:** Pearson correlations among the study variables.

Variable	1	2	3	4	5
Internet Addiction	–				
Psychological Inflexibility	-0.357	–			
Difficulty Identifying Feelings	0.332	-0.684	–		
Difficulty Describing Feelings	0.178	-0.425	0.605	–	
Externally-Oriented Thinking	0.250	-0.023	0.262	0.315	–

**Figure 1:**
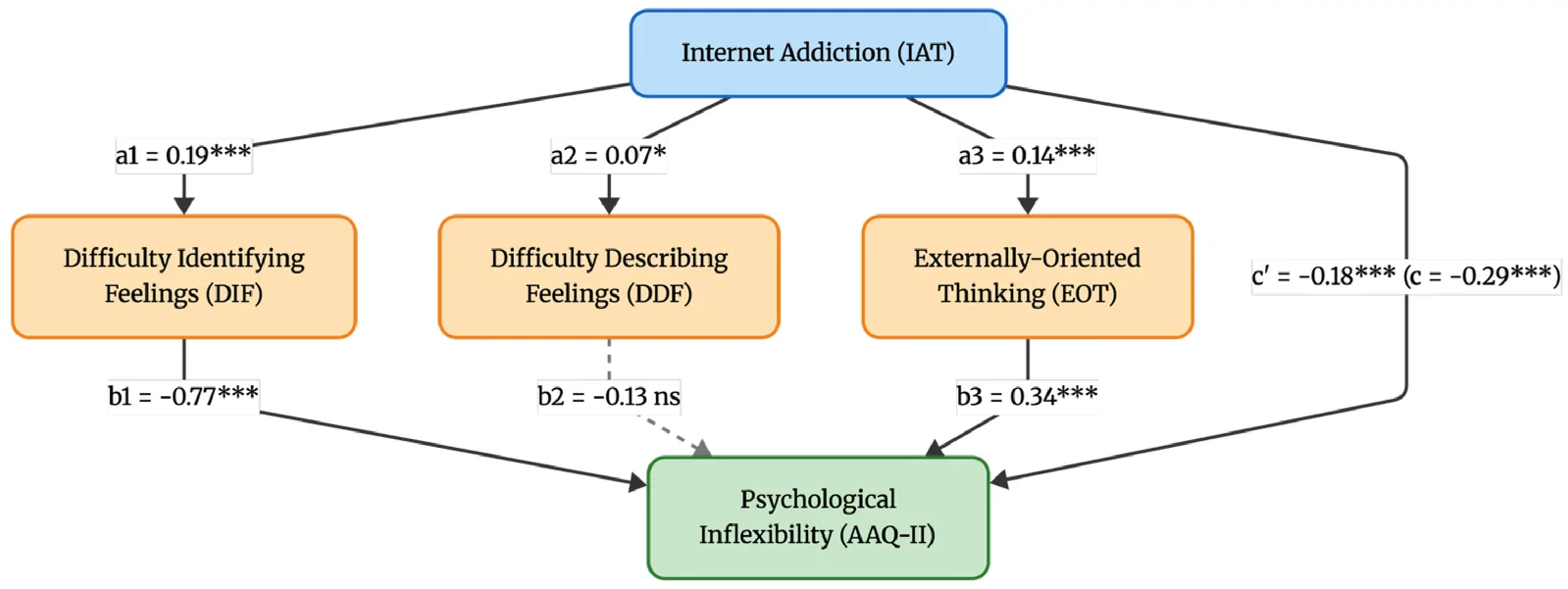
Parallel multiple-mediation model linking internet addiction (IAT) to psychological inflexibility (AAQ−II) via three TAS−20 facets. Path coefficients shown are unstandardized estimates. *p ≤ 0.05, *** p ≤ 0.001.

The parallel mediation analysis was conducted on a final sample of N = 224 subjects, following the exclusion of three cases presenting missing data (**Figure 1**). In the first block of the analysis, concerning the prediction of the mediators by the independent variable (Paths a), internet addiction emerged as a positive and statistically significant predictor of all three tested alexithymia dimensions. Specifically, IA scores were significantly associated with higher levels of DIF (b=0.19, SE = 0.05, t=3.97, p<.001), DDF (b=0.07, SE = 0.03, t=2.17, p=.031), and EOT (b=0.14, SE = 0.04, t=3.86, p<.001).

The overall linear model aiming to predict the distress outcome, represented by psychological inflexibility, was highly significant (F (12,211)=35.81, p<.001) and accounted for 57.0% of the total variance of the construct (R^2^ = .570). Examination of the direct relationships between the mediators and the outcome variable (Paths b) highlighted opposing and differentiated associative trajectories: the DIF dimension showed a negative and statistically significant association with psychological inflexibility (b=−0.77, SE = 0.09, t=−9.02, p<.001), whereas the EOT dimension exhibited a positive and significant link with the same dependent variable (b=0.34, SE = 0.10, t=3.48, p<.001). Conversely, the DDF subscale was not a statistically significant predictor of inflexibility scores (b=−0.13, SE = 0.11, t=−1.19, p=.234). Controlling for the three parallel mediators and the covariates, the direct effect of IA on psychological inflexibility (Path c’) remained statistically significant and negative in sign (b=−0.18, SE = 0.05, t=−3.58, p<.001), indicating a partial mediation process with a statistical suppression, compared to the initial total effect detected in the absence of the mediators (b=−0.29, SE = 0.06, t=−4.65, p<.001).

The significance of the indirect effects was verified through the analysis of confidence intervals generated with 5,000 bootstrap resamples (**Table 4**). The total indirect effect of IA on psychological inflexibility was statistically significant and indicated a small-to-moderate standardized magnitude ( ${\text{β}}_{\text{a}}{\text{β}}_{\text{b}}=-0.146$). Analysis of the individual specific mediation pathways confirmed the existence of two statistically relevant paths with opposite signs: the indirect path mediated by DIF showed a small-to-moderate negative effect ( ${\text{β}}_{\text{a}}{\text{β}}_{\text{b}}=-0.184$), while the indirect path mediated by EOT showed a small positive effect ( ${\text{β}}_{\text{a}}{\text{β}}_{\text{b}}=-0.051$). The specific mediation pathway passing through DDF was not significant and was negligible in magnitude ( ${\text{β}}_{\text{a}}{\text{β}}_{\text{b}}=-0.014$).

**Table 4:** Total and specific indirect effects of internet addiction on psychological inflexibility through alexithymia facets.

Mediation pathway	$b_{a}b_{b}$	$\beta_{a}\beta_{b}$	Boot SE	LLCI	ULCI
Total Indirect Effect	-0.146	-0.148	0.046	-0.234	-0.054
Indirect Effect via DIF	-0.183	-0.185	0.041	-0.263	-0.102
Indirect Effect via DDF	-0.014	-0.015	0.012	-0.041	0.004
Indirect Effect via EOT	0.051	0.052	0.020	0.018	0.094

Bootstrapping 95% CI on 5,000 resamples. ${\text{b}}_{\text{a}}{\text{b}}_{\text{b}}$, Unstandardized Indirect Effect; ${\text{β}}_{\text{a}}{\text{β}}_{\text{b}}$, Completely standardized Indirect Effect; LLCI, Lower Limit Confidence Interval; ULCI, Upper Limit Confidence Interval.

Inspection of the specific contrasts revealed that the path through DIF resulted statistically stronger than that through EOT (Effect = 0.133, BootSE = 0.045, 95% BootCI [0.043, 0.220]).

## 4 Discussion

The primary aim of the current study was to examine how Internet addiction was associated with psychological inflexibility, with particular attention to the differential role of distinct alexithymia facets within young adults. Psychological inflexibility and experiential avoidance are central cognitive-emotional mechanisms in the development and maintenance of Internet addiction (15). Our findings suggest a “pseudo-flexibility” pattern, wherein the total effect of Internet addiction on psychological inflexibility was partially explained by two alexithymic pathways: Difficulty Identifying Feelings and Externally-Oriented Thinking. Interpreted within the I-PACE model, these findings suggest that the interplay between P-level affective vulnerabilities (alexithymia) and E-level deficits in volitional control (psychological inflexibility) does not follow a simple additive logic, but rather produces an asymmetric and opposing pattern mediated by distinct alexithymic facets.

This paradox can be further contextualized within the structural properties of the digital environment: consistent with the A-C level of the I-PACE model, the modern internet may function as a structural scaffolding for emotional avoidance (19), fostering chronic absorption and reduced interoceptive monitoring.

Contrary to our initial hypothesis (H1), the most prominent indirect effect identified in our statistical models is the negative mediation pathway through DIF, which constitutes the primary empirical basis of the “pseudo-flexibility” paradox. While IA scores were positively associated with all alexithymia facets, DIF showed an inverse association with psychological inflexibility. This phenomenon may reflect a compensatory process (53) in which compulsive Internet use is associated with reduced awareness of negative internal states, in line with the self-medication hypothesis (41). Crucially, since DIF involves a marked incapacity to differentiate feelings from bodily sensations (27), respondents may lack the necessary emotional clarity to perceive and self-report the “rigid dominance of psychological reactions” that the Acceptance and Action Questionnaire-II may fail to measure. Consequently, IA may be associated with higher self-reported flexibility, not necessarily reflecting a mature, mindful capacity to navigate distress, but possibly co-occurring with a reduced ability to recognize such internal states - consistent with an anesthetic-like effect of the digital medium. This pattern is consistent with a P-level vulnerability so pervasive that it may interfere with the accurate self-monitoring required for the E-level processes captured by the AAQ-II. However, the reverse pathway should also be considered: individuals with preexisting emotional blindness and reduced interoceptive awareness may be more likely to gravitate toward the Internet as a compensatory scaffold that helps them avoid internal processing demands, consistent with the cyclical reinforcement emphasized by the I-PACE model. (21, 22).

The second significant mediation pathway identified in our study is the positive indirect effect of IA on psychological inflexibility via EOT. This finding suggests that higher Internet addiction scores are associated with a cognitive style characterized by preoccupation with external events and concrete details, alongside greater psychological rigidity. As conceptualized by Bagby et al. (42), EOT reflects an avoidant focus on the external world that limits introspective awareness — functioning as a form of experiential avoidance, consistent with the mechanics of avoidance coping identified as a core correlate of IA severity in adult populations (14). This outward orientation – characterized by preferential attention toward external stimuli at the expense of internal emotional processes (54) – may be associated with reduced opportunities to develop the perspective-taking skills as “self-as-context”. (39).

A pivotal finding of the current study emerges from the statistical contrast between these two significant mediation pathways. The contrast analysis confirms that the absolute magnitude of the negative indirect effect through DIF is significantly stronger than the positive indirect effect mediated by EOT. This disparity suggests that the overall pattern observed in our sample may reflect the combined influence of partially opposing psychological mechanisms, with emotional unawareness potentially overshadowing cognitive rigidity in the self-reported responses.

This asymmetric cross-talk between opposing mediating pathways is consistent with the I-PACE model’s emphasis on the dynamic and mutually interfering nature of P-level and A-C/E-level processes. (20–22).

In contrast to the significant pathways observed via DIF and EOT, the DDF subscale did not emerge as a significant predictor. From a theoretical perspective, Sifneos (27) distinguished between the primary internal incapacity to differentiate feelings (DIF) and the secondary communicative failure to verbalize those states to others (DDF). Our results suggest that psychological inflexibility is more strongly anchored in the initial stage of emotional awareness than in the subsequent stage of verbal reporting (31). Because IA may operate as a “psychic retreat”, the internal confusion characterized by DIF appears more closely associated with patterns consistent with masked psychological rigidity than the interpersonal deficit of describing feelings to an external audience. (30).

The prominent role of interpersonal dynamics in this clinical picture is further corroborated by the significant main effects of our covariates: social difficulties and social avoidance emerged as robust positive predictors of both alexithymia and psychological inflexibility. The unique architecture of the digital environment — characterized by the absence of physical proximity — offers a favorable medium for individuals who struggle with the complexities of face-to-face relationships (55). Consequently, the digital ecosystem may serves as a specific compensatory refuge where individuals with high alexithymic traits appear to regulate their emotions with greater ease (13, 31). Within this context, the reported pseudo-flexibility likely represents a sort of compensatory response to unmet social needs (56). Taken together, these results underscore that the relationship between problematic Internet use and psychological inflexibility should be interpreted within a broader debate on the heterogeneous nature of Internet-related problems and the role of emotional vulnerabilities in shaping maladaptive online behaviors.

Overall, the present findings inform and extend the I-PACE framework by showing that alexithymia is not a unitary vulnerability, but a multidimensional construct with potentially distinct effects on the transition from problematic Internet use to psychological inflexibility. In particular, DIF and EOT appear to contribute in opposite ways to this process, whereas DDF shows a weaker role, suggesting that the translation of emotional vulnerabilities into executive-control difficulties may depend on the specific alexithymic facet involved.

Finally, the present findings suggest that prevention and intervention programs for problematic Internet use may benefit from targeting emotional awareness and psychological flexibility, especially in young adults with prominent alexithymic traits. Clinicians should also be cautious in relying exclusively on self-report flexibility measures such as the AAQ-II when screening this population, because self-report scores may not fully capture rigidity in individuals with marked difficulty identifying or describing emotions. A multimethod assessment combining questionnaires with clinical interview and, when feasible, behavioral indicators may therefore improve case formulation and treatment planning.

### 4.1 Limitations and future directions

The findings of the current study must be interpreted considering several methodological limitations. First, the cross-sectional design precludes any definitive conclusions regarding the causal direction, and the observed associations may reflect reciprocal or cyclical processes rather than a single directional pathway (57); although the proposed mediation model was theoretically grounded in the I-PACE framework, the observed indirect effects should therefore be interpreted as statistical associations rather than evidence of temporal or causal mechanisms.

Second, the exclusive reliance on self-report instruments - such as the AAQ-II – maybe particularly problematic in a sample characterized by high DIF, because individuals with elevated alexithymic traits may struggle to evaluate their own emotional deficits (54) and psychological reactions.

Third, the relatively low variance explained for the EOT and DDF pathways suggests that other unmeasured factors, such as underlying psychopathology (19) or negative childhood experiences and trauma (28, 58), likely contribute significantly to the development of both alexithymia and addiction.

Fourth, the conceptual status of Internet Addiction remains debated, and problematic Internet use is a heterogeneous phenomenon that may encompass different forms of online behavior with partially distinct motivational and emotional mechanisms.

Finally, the predominantly female composition of the sample and the exclusive inclusion of young adults recruited through convenience sampling may limit generalizability, especially given evidence that gender can shape patterns of Internet use and PIU-related symptoms (59, 60). Future research should therefore adopt longitudinal designs, combine self-report with behavioral and clinician-rated measures, examine whether the present mediation model generalizes across specific PIU subtypes, incorporate additional psychological and developmental risk factors, and test the robustness of these pathways in gender-balanced and culturally diverse samples.

### 4.2 Conclusion

This study highlights a complex pattern linking Internet addiction and psychological inflexibility through the distinct facets of alexithymia. Within an I-PACE framework, our findings suggest that affective vulnerabilities and executive-control difficulties may be simultaneously implicated, with Difficulty Identifying Feelings emerging as the strongest indirect pathway and Externally-Oriented Thinking showing an opposing but weaker contribution, thereby underscoring the differential role of alexithymia facets. Overall, the results indicate that the observed association between Internet addiction and psychological inflexibility may reflect a form of apparent flexibility rather than a genuine adaptive capacity, suggesting that interventions targeting emotional awareness and psychological flexibility may be particularly relevant for problematic Internet use, although future work is needed to determine whether these pathways differ across specific PIU subtypes and gender groups.

## Data Availability

The original contributions presented in the study are included in the article/supplementary material. Further inquiries can be directed to the corresponding author.
